# Inhibition of APOE potentiates immune checkpoint therapy for cancer

**DOI:** 10.7150/ijbs.70117

**Published:** 2022-08-15

**Authors:** Bingqing Hui, Chen Lu, Haiyang Li, Xiaopei Hao, Hanyuan Liu, Danping Zhuo, Qian Wang, Zhouxiao Li, Li Liu, Xuehao Wang, Yanhong Gu, Weiwei Tang

**Affiliations:** 1 Department of Oncology, The First Affiliated Hospital of Nanjing Medical University, Nanjing, Jiangsu, China.; 2 Department of General Surgery, The First Affiliated Hospital of Nanjing Medical University, Nanjing, Jiangsu, China.; 3 Department of General Surgery, Nanjing First Hospital, Nanjing Medical University, Nanjing, Jiangsu, China.; 4 Hepatobiliary/Liver Transplantation Center, The First Affiliated Hospital of Nanjing Medical University, Key Laboratory of Living Donor Transplantation, Chinese Academy of Medical Sciences, Nanjing, Jiangsu, China.; 5 First Teaching Hospital of Tianjin University of Traditional Chinese Medicine, Tianjin, China.; 6 State Key Laboratory of Modern Chinese Medicine, Tianjin University of Traditional Chinese Medicine, Tianjin, China.; 7 Research Unit Analytical Pathology, Helmholtz Zentrum München, German Research Center for Environmental Health (GmbH), Neuherberg, Germany.; 8 Department of plastic and hand surgery, University Hospital Munich, Campus Innenstadt, Germany.

**Keywords:** single-cell RNA sequencing, APOE, PD-1, TIGIT, macrophage

## Abstract

Checkpoint immunotherapy is capable of unleashing T cells for controlling tumor, whereas it is destroyed by immunosuppressive myeloid cell. Apoprotein E (APOE) refers to a ligand in terms of the members of low-density lipoprotein (LDL) receptor family for mediating Apoprotein B-involving atherogenic lipoprotein clearance. Besides, tumor-infiltration macrophage can express APOE. The present study reported Apoe^-/-^ mice to exhibit higher resistance toward the development of three types of carcinomas as compared with mice with wild type and to have greater responses to αPD-1 (anti-PD-1) immunotherapy. Moreover, treatment by exploiting APOE inhibitor (COG 133TFA, αAPOE) was capable of curbing tumor development and fostering regression if in combination of αPD-1. According to single-cell RNA sequencing (scRNA-seq), Apoe deletion was correlated with the decline of C1QC^+^ and CCR2^+^ macrophage within tumor infiltration, and mass spectrometry results noticeably showed down-regulated the number of M2 macrophages as well. Furthermore, APOE expression in cancer patients resistant to αPD-1 treatment significantly exceeded that in the sensitive group. For this reason, APOE is likely to be targeted for modifying tumor macrophage infiltrate and augmenting checkpoint immunotherapy.

## Introduction

To evade immune surveillance, carcinoma cells mask their immunogenicity, while inducing one environment at a microscale suppressing immunization response in an active manner. Inhibitory mechanism can exert direct influence on T cell response through involvement in immunization checkpoint (e.g., programmed death 1 (PD-1) as well as cytotoxic T lymphocyt-related antigen 4 (CTLA-4) 1, 2. Tumors also use macrophages to actively inhibit the anti-tumor T cell response 3. According to recent high-dimensional profiling studies, especially single-cell RNA sequencing (scRNA-seq), tumor-infiltration macrophage cell exhibit high heterogeneity and are likely to cover subdivided sets that are immunosuppressive and immunostimulatory 4, 5.

Apolipoprotein E (APOE) refers to a primary brain cholesterol carrier, which affects a wide range of common cell-related process (e.g., neuroinflammation, amyloid β (Aβ) degradation and clearance, synaptogenesis, membrane repair and remodeling, and neuronal growth) 6. Recently, APOE has been reported associated with cancer. Previous study showed that APOE was highly expressed in gastric cancer, contributing to shorter survival. In particular, APOE was closely related to muscular invasion, and may be a biomarker predicting muscular invasion of gastric cancer 7. Zheng Peiming et al identified that APOE was a highly specific and effective protein in M2 macrophage-derived exosomes. Exosomes derived from M2 macrophages mediate intercellular transfer of PI3K-Akt signaling pathway activated by APOE in recipient gastric cancer cells, thereby reshaping the migration of cytoskeleton 8.

However, the effect exerted by APOE within tumor immunization responses especially in tumor-associated macrophages (TAM) is still not tackled down. In this study, we reported Apoe^-/-^ mice to exhibit higher resistance toward the development of three types of carcinomas including gastric carcinoma (GC), colorectal carcinoma (CRC), and hepatocellular carcinoma (HCC) as compared with mice with wild type (WT) and to have greater responses to αPD-1 (anti-PD-1) immunotherapy. Moreover, treatment by exploiting APOE inhibitor (αAPOE) was capable of curbing tumor development and fostering regression if in combination of αPD-1.

## Materials and methods

### Single-cell RNA transcriptome analysis

This study analyzed the expression of Apoe in CRC, GC and HCC tissues with scRNA-seq, as derived from Sijin Cheng' research 9.Tumors from WT and Apoe^-/-^ MC38 C57BL/6 mice were used for scRNA-seq by ourselves.The software packages used for specific analysis are listed in Table S1.

### Cases and tissue specimen collection

Based on Declaration of Helsinki, we made the informing process with respect to the present study. Human 30 carcinoma tissues around received the collection from cases having undergone surgeries under informed consent from the First Affiliated Hospital of Nanjing Medical University and Nanjing First Hospital. Moreover, 19 samples before αPD-1 treatment were obtained from above hospitals. The collection of human specimens gained the approval from the Medical Ethics Committee of Nanjing Medical University. The overall samples received the efficiently collection after being removed from the body. They were immediately frozen in liquid nitrogen and then stored at 80 °C for subsequent use. The mentioned tumor specimens were confirmed and classified by experienced clinicians.

### Cancer cell culture

The Cell Bank of Type Culture Collection (Chinese Academy of Sciences, China) offered mice MC38-CRC, MFC-GC, and Hepa1-6-HCC cells, human CRC cells (HCT116), human GC cells (HGC27), human HCC cells (LM3) cultured with RPMI 1640 medium (BI, USA) supplemented by 10% fetal bovine serum (FBS) (Gibco, USA) at 37 °C in a 5% CO2 chamber.

### Primary culture of THP-1

THP-1 cells were cultured in RPMI 1640 medium (BI, USA) supplemented by 10% fetal bovine serum (FBS) (Gibco, USA) and100 nM Phorbol-12-myristate-13-acetate (PMA, sigma aldrich,USA) in 5% CO2 at 37 °C. THP-1 cells were harvested after 2 days of PMA-mediated macrophage differentiation. In TAM stimulation experiments, THP-1 cells were treated with cancer cell culture supernatant in RPMI 1640 medium for 2 days, which produced what we call TAM. TAM cells were plated at a density of 1×10^6^ in a 6-well plate and then treated with PBS and αAPOE (COG133TFA, sigma aldrich, USA) for 24 hours at 37 °C, respectively.

### Cell counting kit-8 proliferation experiment

Cancer cells were treated with supernatant derived from TAM, which received PBS or αAPOE treatment. Then cancer cells were seeded in 96 wells and then administrated with 10 μl of CCK8 solution (Ribobio, China) when cultured at 0h, 24h, 48h, and 72h, separately. Subsequently, cells absorbance at the respective time received the analysis at 450 nM by microplate reading element by conforming to the producer's instructions (Synergy4, USA).

### Transwell invasion assays

Cancer cells were treated with supernatant derived from TAM, which received PBS or αAPOE treatment. Cancer cells were then subjected to the inoculation process in the upper chamber with 200 μl of serum-free RPMI 1640 medium. Transwell chambers (Corning, USA) were paved with matrigel mixture (BD Biosciences, USA) to enable invasion assays. RPMI 1640 medium and 10% FBS were introduced into the basal compartment as cancer cell chemoattractant. After 24 h of culture, the upper chamber was subjected to the fixation process and then stained with crystal violet (Kaigen, China) for 15 min. For visualization, cell lines were photographed and counted in three fields of view.

### Scratch wound experiment

Cancer cells were treated with supernatant derived from TAM, which received PBS or αAPOE treatment. Under the cell confluence reaching about 90%, wounds received the creation with a 200 µl pipette tip, and the cells received the rinsing process with medium for the removal of free-floating cells and debris. Medium received the addition, and the culturing plates underwent the incubation at 37 °C. Wound healing received the survey at various points. Furthermore, representing scrape lines were captured.

### Quantitative reverse transcription polymerase reaction (qRT-PCR)

Given the producer's protocol, total RNAs from TAM cells received the isolation based on TRIzol reagent (Invitrogen, USA). Based on reverse transcription kit (Takara, Japan), cDNA received the synthesis for mRNA; based on RiboBio reverse transcription kit (China). GAPDH was used to normalize the mRNA expressing level levels.

### Immunohistochemistry

The paraffin-embedded sections were dewaxed and rehydrated. 3% hydrogen peroxide was used to block peroxidase activity. Sections were incubated throughout the night with primary antibody (human APOE, PD-1, TIGIT, abcam, UK, mouse C1QC, CCR2, CD206, CD86, abcam, UK) at 4 °C. Next, the biotinylated secondary antibody was adopted to treat tissue sections and then incubated with streptavidin-horseradish peroxidase complex (Santa Cruz Biotechnology Inc., USA).

### Mice model

The animal management committee of Nanjing Medical University approved the animal experiment, and all experiment procedures and animal caring abided by the institutional ethics directions for animals-related experimental processes. The injection of MC38, MFC and Hepa1-6 cells was made into Apoe^+/+^ (n=4) and Apoe^-/-^ (n=4) C57BL/6 mice (GemPharmatech, China), respectively. Carcinoma transplanted tumor model mice fell to four groups, i.e., control, αPD-1/αTIGIT group (αPD-1, bioxcell, USA; αTIGIT, bioxcell, USA), COG133TFA (αAPOE, sigma aldrich, USA) group, αPD-1/αTIGIT and COG133TFA combined group (αAPOE+αPD-1/αTIGIT), with 4 mice in the respective group. The four groups of mice were treated by complying with the corresponding groups. To be specific, αPD-1/αTIGIT group received 6.6mg/kg intraperitoneal injection on the eighth day, and once per three days thereafter; COG133TFA group underwent the intraperitoneal injection of 1 mg/kg on the second day and once per five days thereafter; αPD-1/αTIGIT and COG133TFA group received the intraperitoneal injection of COG133TFA 1 mg/kg on the second day and once per five days thereafter, as well as the intraperitoneal injection of αPD-1/αTIGIT 6.6mg/kg on the eighth day and once per three days thereafter; Control received the intraperitoneal injection of PBS 100ul/ mouse on the second day and once per five days thereafter.The activity, spirit and diet of the mice were observed every day before and after the experiment. The long diameter of the tumor A(mm) and the short diameter B(mm) were measured with vernier calipers every 4 days, and the tumor volume (V) was calculated by V=AB^2^/2 to obtain the tumor growth curve.

### Mass spectrometry

The tissue samples originated from Apoe^-/-^, Apoe^+/+^, Apoe^+/+^+αPD-1, Apoe^-/--^+αPD-1 mice, respectively. The treatment method of mouse tissue was referred to Miltenyi Biotec mouse tumor isolation Kit. Percoll removed impurities and split red. CyTOF staining steps included 194Pt staining → Fc block → surface antibody staining → overnight DNA staining → intracellular antibody staining → data collection on computer.

### Statistical analysis

The analyses were mainly performed with GraphPad Prism8 (GraphPad Prism, USA) and p-value < 0.05 was distinguished for exhibiting statistics-related significance.

## Results

### Apoe was over-expressed in TAM of cancer tissues based on scRNA-seq database

**Figure 1:**
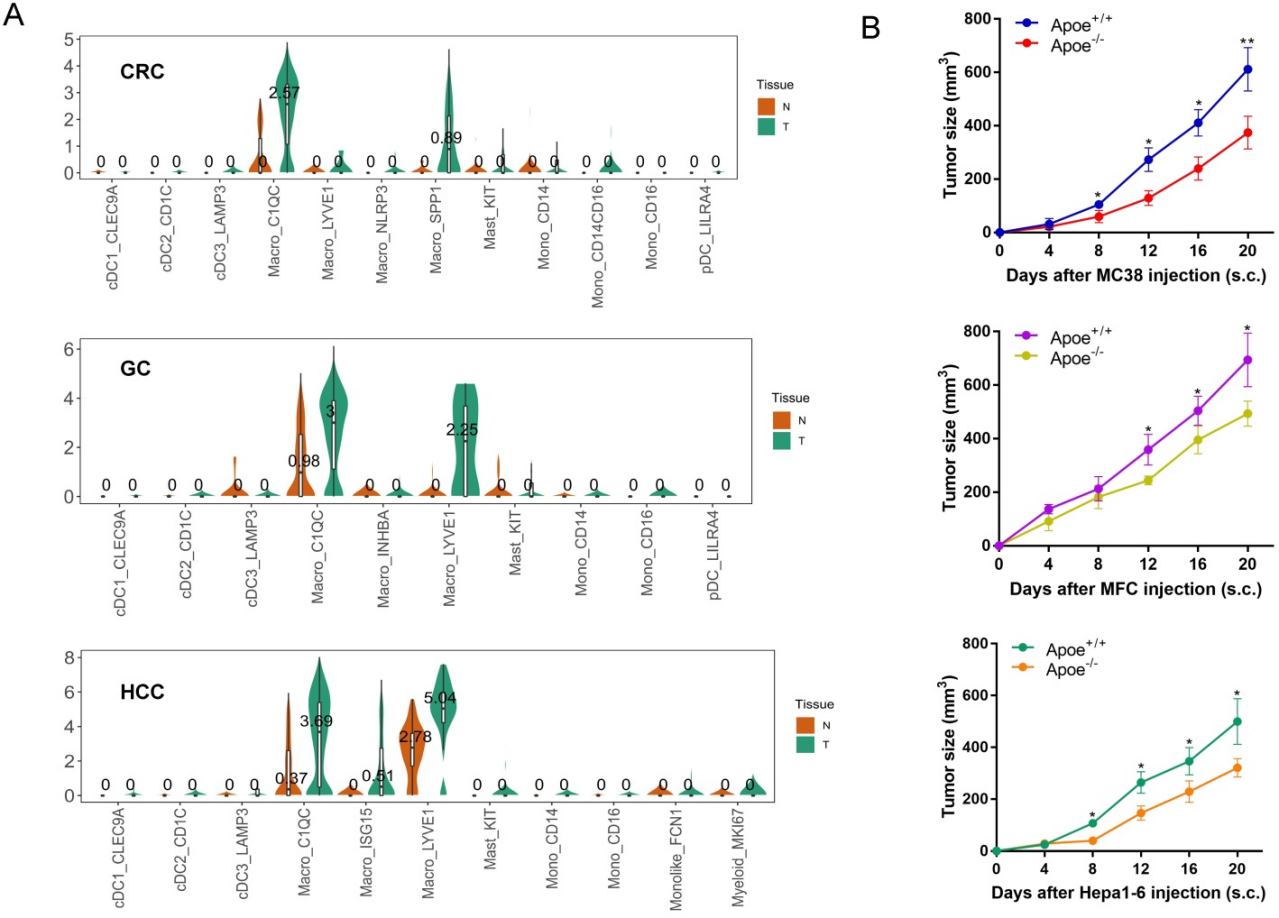
** Apoe was over-expressed in TAM of cancer tissues based on scRNA-seq database. (A)** scRNA-seq results showing Apoe expression in CRC, GC, and HCC. **(B)** Tumor growth in Apoe^+/+^ and Apoe^-/-^ mice injected subcutaneously with MC38, MFC, Hepa1-6, respectively.*p < 0.05, **p < 0.01.

This study analyzed the expression of Apoe in CRC, GC and HCC with scRNA-seq, as derived from Sijin Cheng' research 9 and results showed that TAM in cancer tissues expressed higher Apoe compared with tissues adjacent to carcinoma in these tumors. In particular, the expression of Apoe in the C1QC ^+^ macrophages of carcinoma tissues was significantly higher than that of adjacent tissues in three types of carcinomas (Figure 1A). The above results aroused our curiosity to explore why Apoe was specifically upregulated in TAM of cancers.

### Apoe deficiency inhibited tumor growth and reshaped the tumor immune microenvironment based on mass spectrometry in CRC

**Figure 2:**
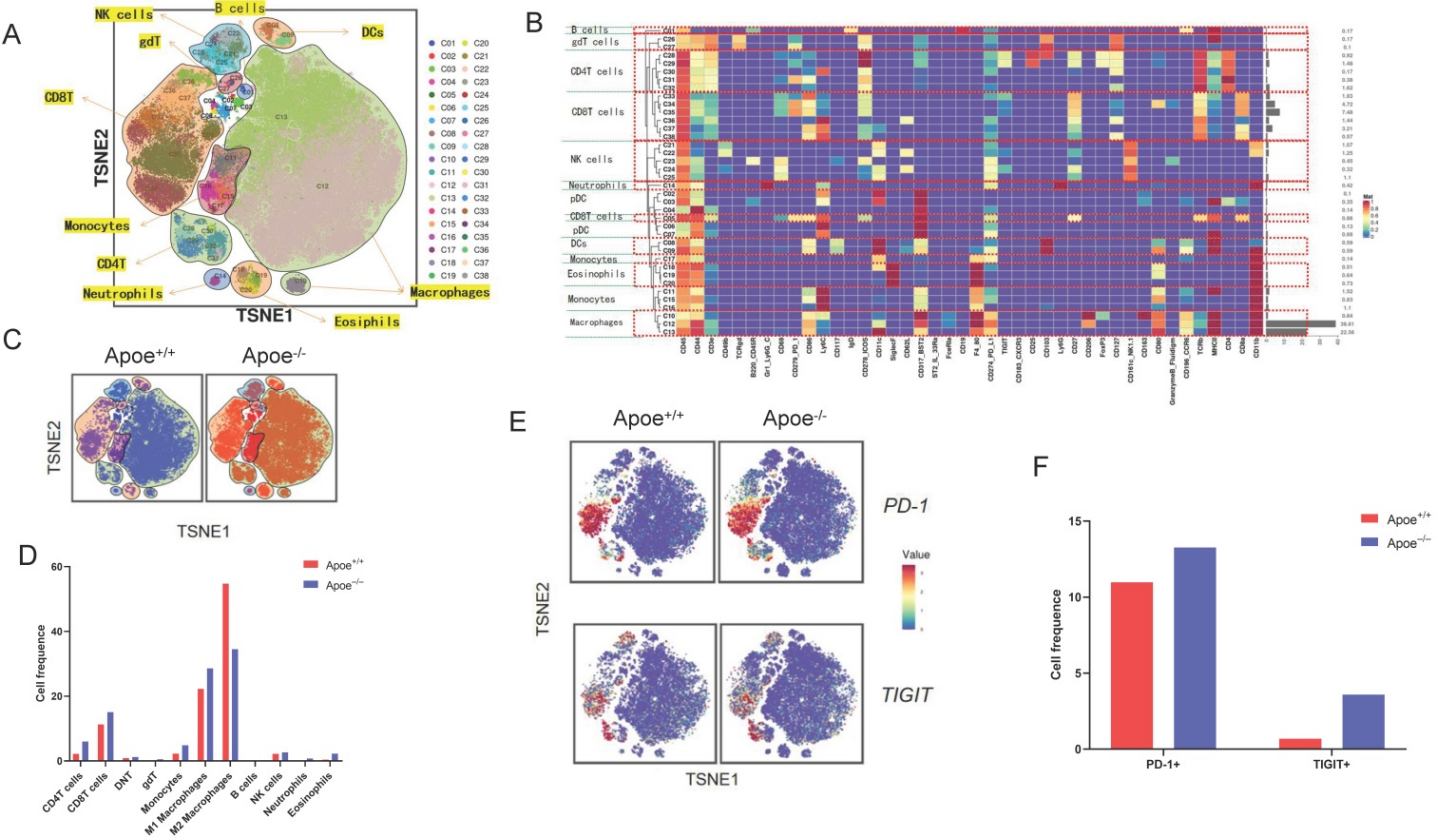
** Apoe deficiency inhibited tumor growth and reshaped the immune microenvironment based on mass spectrometry. (A)** TSNE plot showing definition of 38 subgroups by mass spectrometry flow cytometry in Apoe^-/-^ mice and WT group. **(B)** Cell clustering definition. **(C)** TSNE plot showing cell clustering in Apoe^-/-^ mice group and WT group injected subcutaneously with MC38. **(D)** The histogram showing the number of each cell group in different groups by mass spectrometry flow cytometry. **(E)** TSNE plot showing PD-1 and TIGIT expression in Apoe^-/-^ mice group and WT group. **(F)** The histogram showing the number of PD-1^+^ and TIGIT^+^ cell cluster in different groups.

For addressing the possible effect exerted by Apoe on immunization responses to tumor, we injected cells (MC38-CRC, MFC-GC, and Hepa1-6-HCC) in Apoe^+/+^ and Apoe^-/-^ C57BL/6 mice, respectively. MC38, MFC, and Hepa1-6 cells tended to grow in Apoe^+/+^ mice, whereas they showed the consistent attenuation in Apoe^-/-^ mice (Figure 1B). Subsequently, MC38 was adopted as an example to more specifically explore the effect of Apoe knockout on the tumor immune microenvironment. We tested the immunization infiltration of MC38 tumor within Apoe^+/+^ and Apoe^-/-^ mice 20 days after tumors were injected based on mass spectrometry. We isolated CD45^+^ immune cells from their respective tissues (Figure S1). All samples showed clustering and subgroup annotation of CD45*^+^* immune cells. There were 38 cell clusters in total, and the respective cell clusters were defined based on the specific markers of the respective cell types (Figure 2A-2B, Figure S2). As indicated from the result, the relative proportion of M2 macrophages was significantly down-regulated in the Apoe^-/-^ mice, while the M1 macrophages were up-regulated as compared with Apoe^+/+^ mice (Figure 2C-2D), thereby demonstrating that Apoe deficiency is likely to affect myeloid infiltrate. In addition, it was revealed that monocytes, NK cells, CD4^+^ T cells and CD8^+^ T cells were up-regulated in Apoe^-/-^ mice (Figure 2C-2D). Interestingly, we found that the expression of PD-1^+^ and TIGIT ^+^ cells increased significantly in Apoe^-/-^ mice (Figure 2E-2F). These evidence demonstrated that Apoe knockout might have a function to enhance the sensitivity of checkpoint immunotherapy in CRC.

### αPD-1/αTIGIT enhanced the antitumor effect in Apoe^-/-^ mice

**Figure 3:**
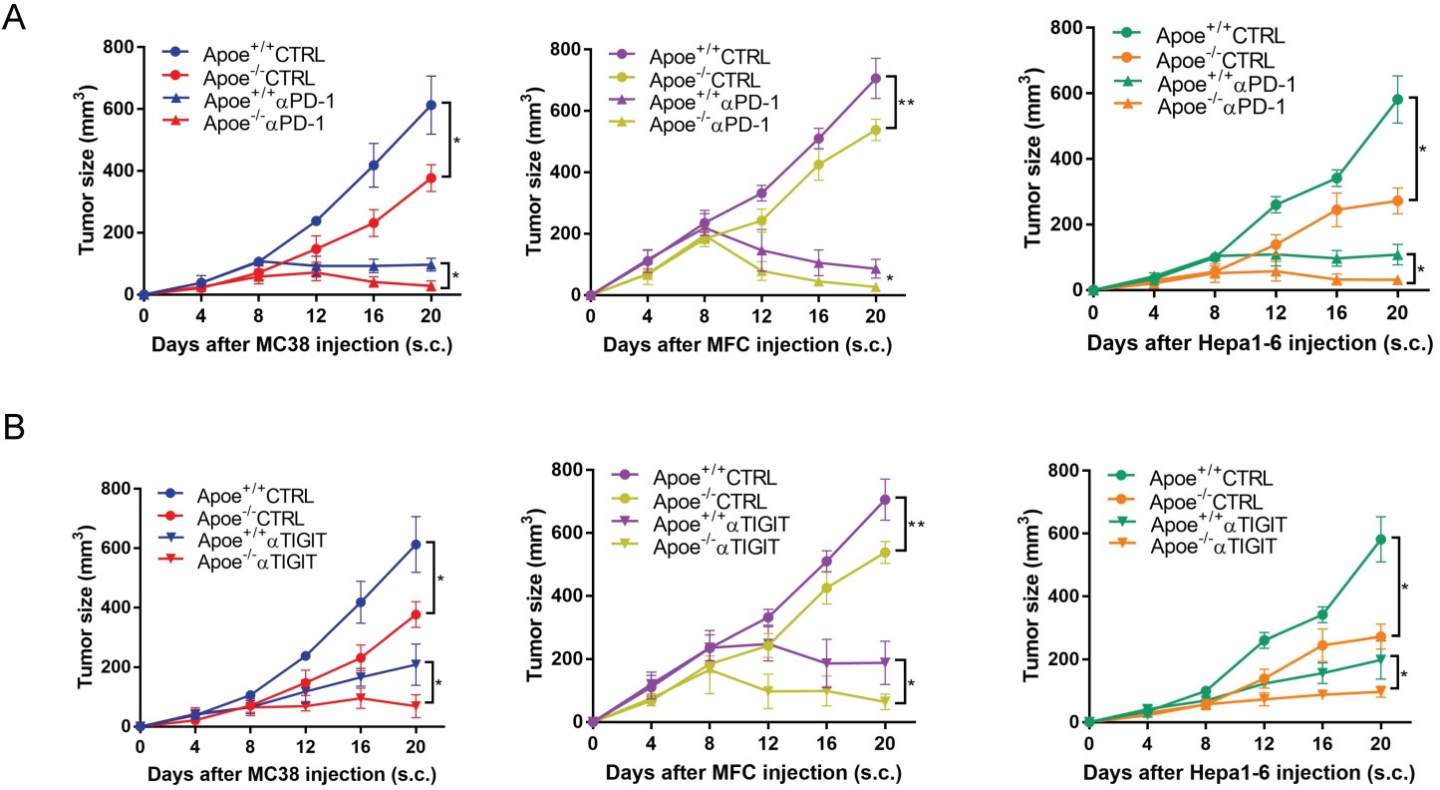
** αPD-1/αTIGIT enhanced the antitumor effect in Apoe^-/-^ mice. (A-B)** Experimental setup of the αPD-1/αTIGIT treatment in Apoe^+/+^ and Apoe^-/-^ mice injected subcutaneously with MC38, MFC, Hepa1-6, respectively. Treatment started at day 8 (αPD-1/αTIGIT). *p < 0.05, **p < 0.01.

**Figure 4:**
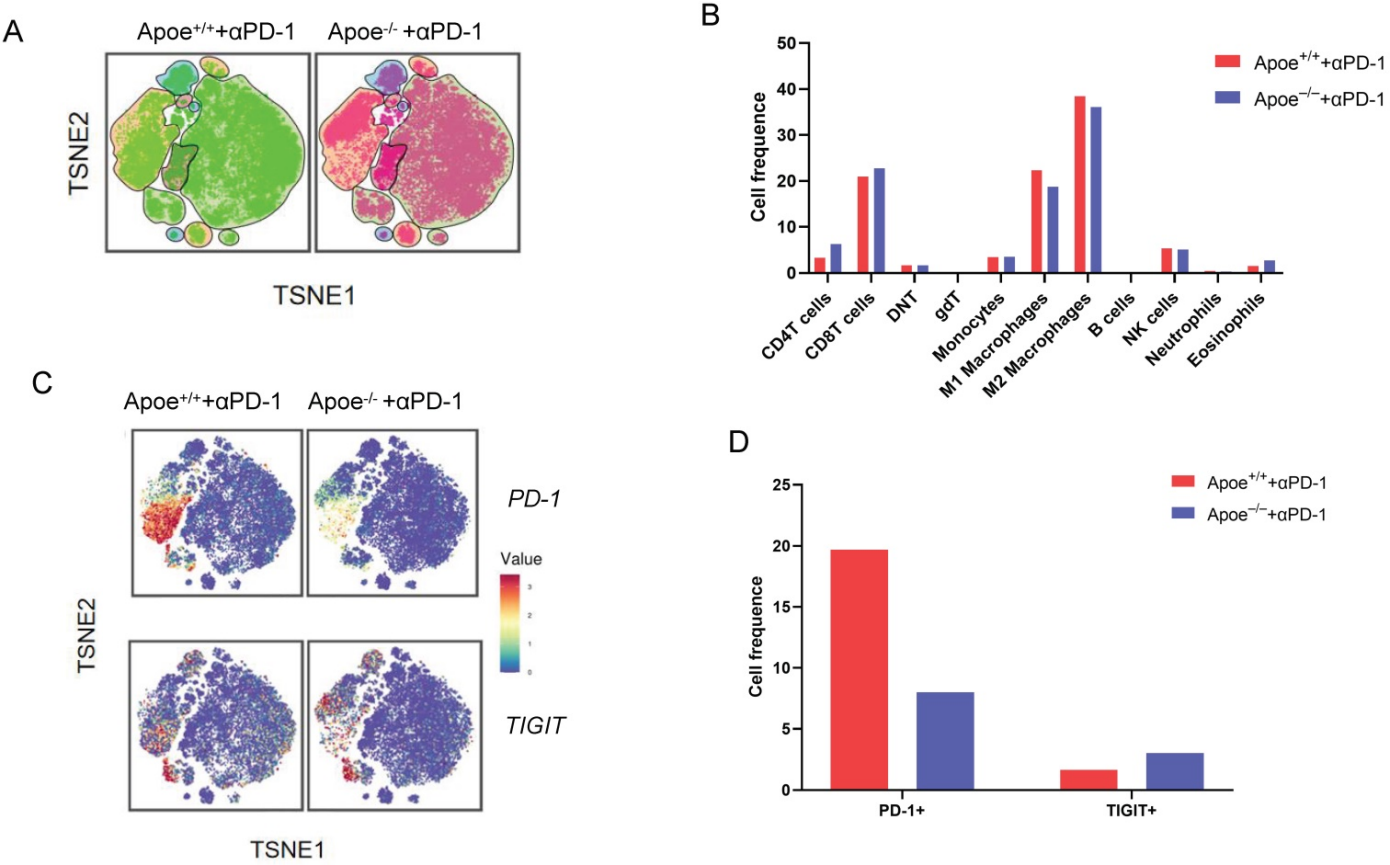
** Results of mass spectrometry in Apoe^-/-^ mice with αPD-1. (A)** TSNE plot showing cell clustering in Apoe^-/-^ mice +αPD-1group and WT mice +αPD-1group. **(B)** The histogram showing the number of each cell group in different groups by mass spectrometry flow cytometry. **(C)** TSNE plot showing PD-1 and TIGIT expression in Apoe^-/-^ mice +αPD-1group and wild type mice +anti-PD-1group. **(D)** The histogram showing the number of PD-1^+^ and TIGIT^+^ cell cluster in different groups.

Based on the above results of mass spectrometry that Apoe deficiency enhanced the expression of PD-1 and TIGIT in CRC, we tried to treat cancers combination with these two antibodies (αPD-1/αTIGIT) in three types of cancer (CRC, GC and HCC). Accordingly, this study selected a scheme to achieve αPD-1/αTIGIT administration, and the treatment was initiated at a late time point (on day 8 after injection of tumor cells). To our excitement, under this treatment protocol, the degree of tumor (MC38, MFC and Hepa1-6) reduction in the Apoe^+/+^ mice was significantly lower than that in Apoe^-/-^ mice (Figure 3A-3B). However, the anti-tumor results of the use of αPD-1outperformed those of αTIGIT. Subsequently, the effect exerted by Apoe^-/-^ mice with αPD-1 on the tumor-immune infiltrate was described by mass spectrometry with MC38 tumor models. Results showed that the relative proportion of CD8^+^ T cells, CD4^+^ T cells were up-regulated while M2 macrophages was reduced in the Apoe^-/-^ +αPD-1mice as compared with Apoe^+/+^+αPD-1 mice (Figure 4A-4B). In addition, we found that the expression of PD-1^+^ cells increased significantly while TIGIT^+^ cells decreased in Apoe^-/-^+αPD-1 mice (Figure 4C-4D). These results revealed that Apoe deficiency might enhance the sensitivity of αPD-1 in cancers.

### Apoe deficiency decreased C1QC^+^ and CCR2^+^ macrophages in cancers based on scRNA-seq

**Figure 5:**
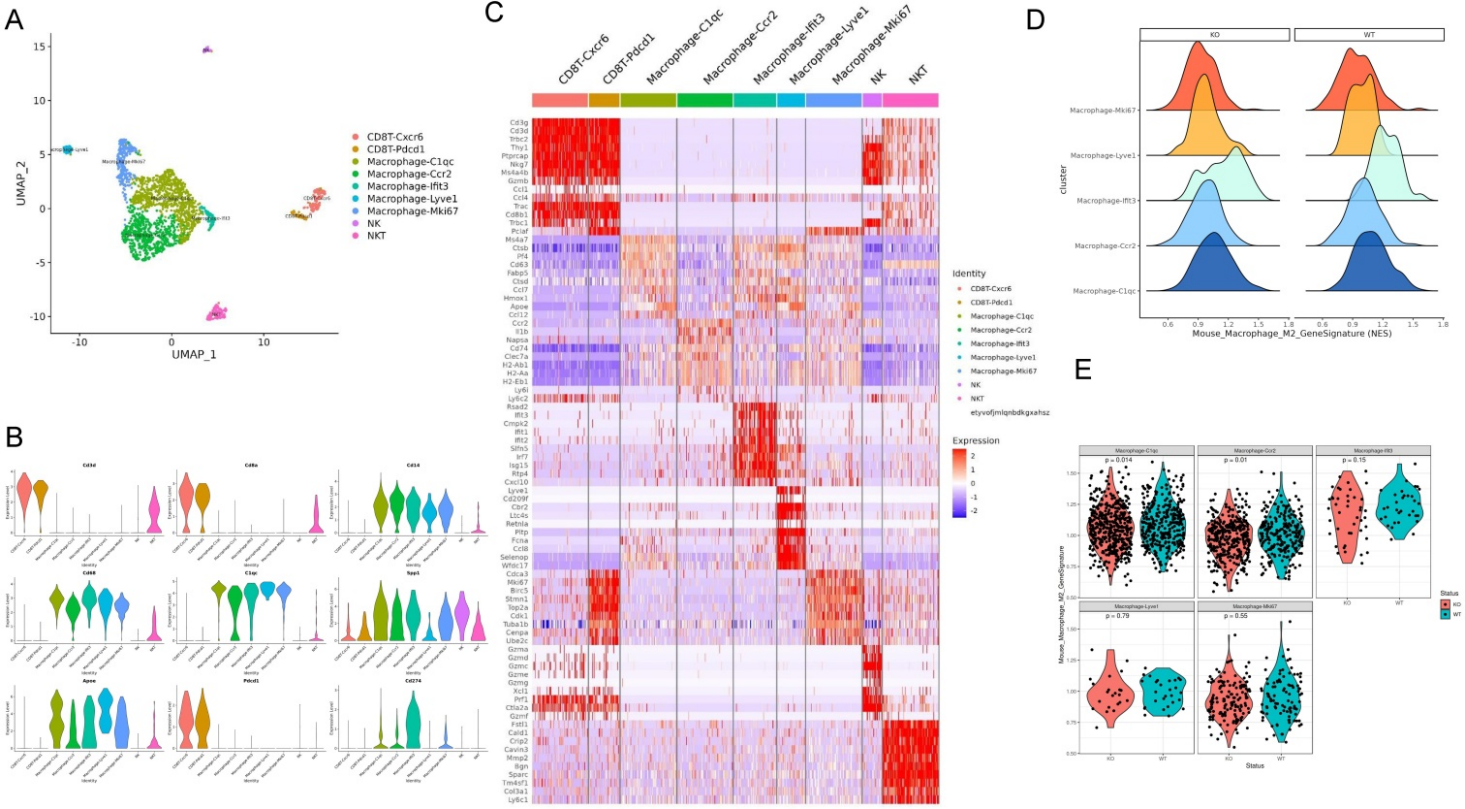
** Apoe deficiency decreased C1QC^+^ and CCR2^+^ macrophages in CRC based on scRNA-seq. (A)** UMAP plot showing 9 clusters of immune cells. **(B)** The violin figure showing the expression level of specific markers in each cell type. **(C)** Heat map showing the expression level of specific markers in each cell type. **(D)** COATES analysis showing the expression difference in macrophage cell clusters. **(E)** The violin diagram showing the macrophage cell clusters different expressions in Apoe^-/-^ mice group and WT group.

For defining the effect of Apoe deficiency on tumor-immunization infiltration at higher resolutions, this study investigated immune cells in Apoe^+/+^ and Apoe^-/-^ MC38 tumor mice based on scRNA-seq. We classified live cells from MC38 tumor 20 days after tumors were injected. Unsupervised clustering by Uniform Manifold Approximation and Projection (UMAP) identified 9 clusters in immune cells (Figure 5A). The respective cell type has unique maker genes (Figure 5B-5C). For instance, CD8^+^ CXCR6^+^ T cell cluster expressed T cell markers (CD3, CD8 and GZMB), while NK cell cluster expressed NKG7. On the whole, 5 macrophage cell clusters (i.e., C1QC, CCR2, IFIT3, LYVE1 and MKI67) were identified (Figure 5A-5C). For a further clarification of the variations of macrophages in the Apoe^-/-^ mice and the control group, we analyzed them by COATES analysis. As revealed from the results, Apoe deletion was correlated with a decline of C1QC ^+^ and CCR2^+^ macrophages in tumor infiltration (Figure 5D-5E). In addition, immunohistochemistry was used to confirm that the expression of C1QC and CCR2 in tissues of the three cancer models with Apoe^-/-^ mice were significantly decreased (Figure S3A). Interestingly, the expression of CD206, CD86, CCR2 and C1QC expression in the Apoe^-/-^ mice non-cancer model compared with Apoe^+/+^ mice was also decreased, indicating that changes in macrophages in the spleen and TAM might be consistent (Figure S3B).

### Inhibition of Apoe in TAM reduced the proliferation, invasion and migration of cancer cells *in vitro*

**Figure 6:**
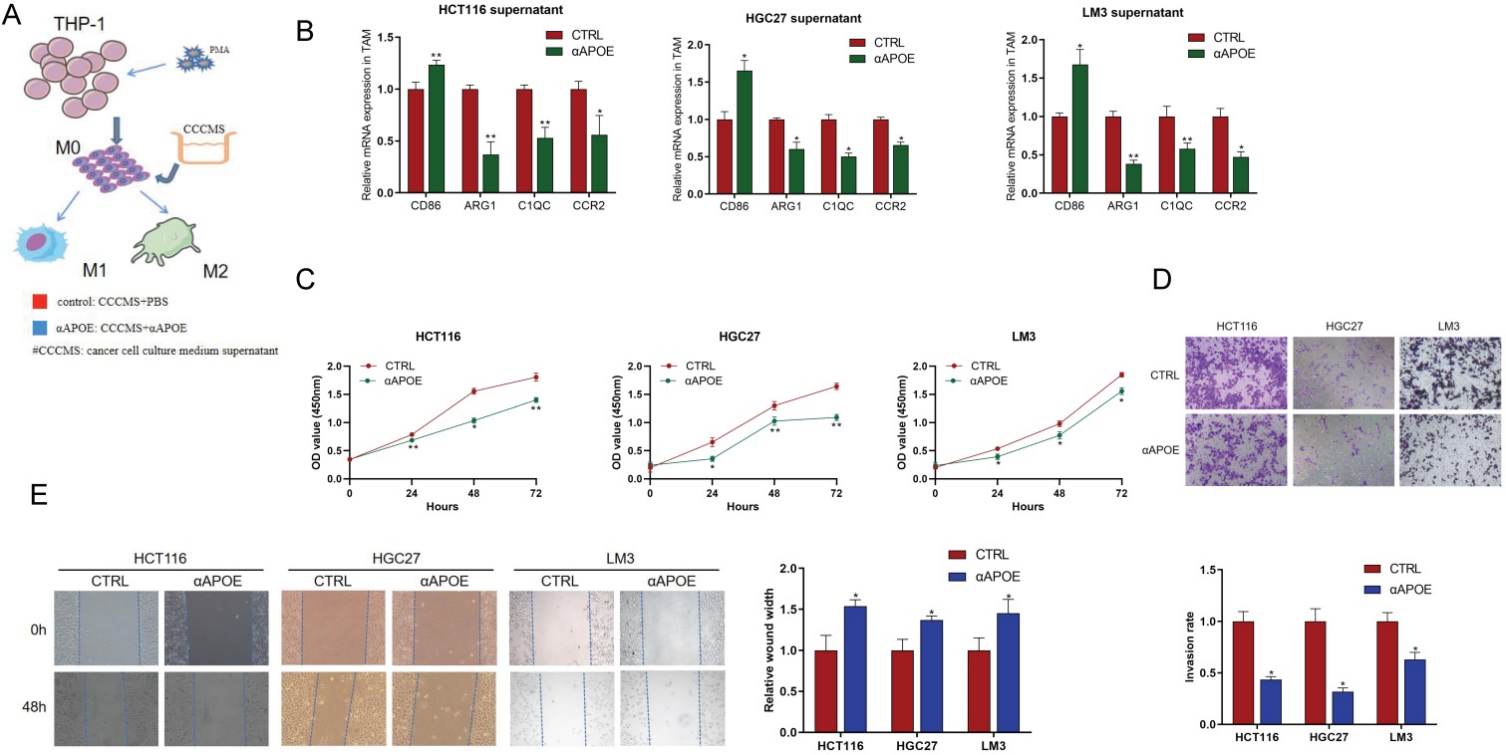
** Apoe deficiency decreased C1QC^+^ and CCR2^+^ macrophages in cancers *in vitro*. (A)** Schematic diagram of THP-1 cells induced into TAM cells. **(B)** qRT-PCR results of M1/M2 phenotype gene expression in the αAPOE and control group after of cancer supernatant to TAM cells activation. **(C)** CCK8 assay of cancer cells with TAM supernatant in different groups. **(D)** Transwell assay of cancer cells with TAM supernatant in different groups. **(E)** Scratch assay of cancer cells with TAM supernatant in different groups.*p < 0.05, **p < 0.01, ***p < 0.001.

In order to further verify the scRNA-seq results of Apoe deficiency decreasing C1QC^+^ and CCR2^+^ macrophages, we conducted in-depth verification. We attempted to use APOE inhibitor (αAPOE, COG 133TFA, competing with the APOE holoprotein for binding the low-density lipoprotein (LDL) receptor) to test its therapeutic potential against carcinomas (Figure 6A). qRT-PCR results showed that CD86 expression of M1 phenotype marker in the αAPOE group was significantly higher than that in the control group after the addition of cancer cell (HCT116 -CRC, HGC27-GC, LM3-HCC) supernatant to TAM cells, while the expression of C1QC,CCR2, and ARG1 of M2 phenotype was significantly lower than that in the control group (Figure 6B). In addition, we stimulated cancer cells with TAM supernatant and found that macrophages in the αAPOE group significantly inhibited the proliferation, invasion and migration of cancer cells compared with the control group by CCK8, transwell and scratch assay (Figure 6C-6E). These results indicated that inhibition of Apoe in TAM reduced the proliferation, invasion and migration of cancer cells.

### The combination of αAPOE and αPD-1/αTIGIT was an effective treatment for cancer

**Figure 7:**
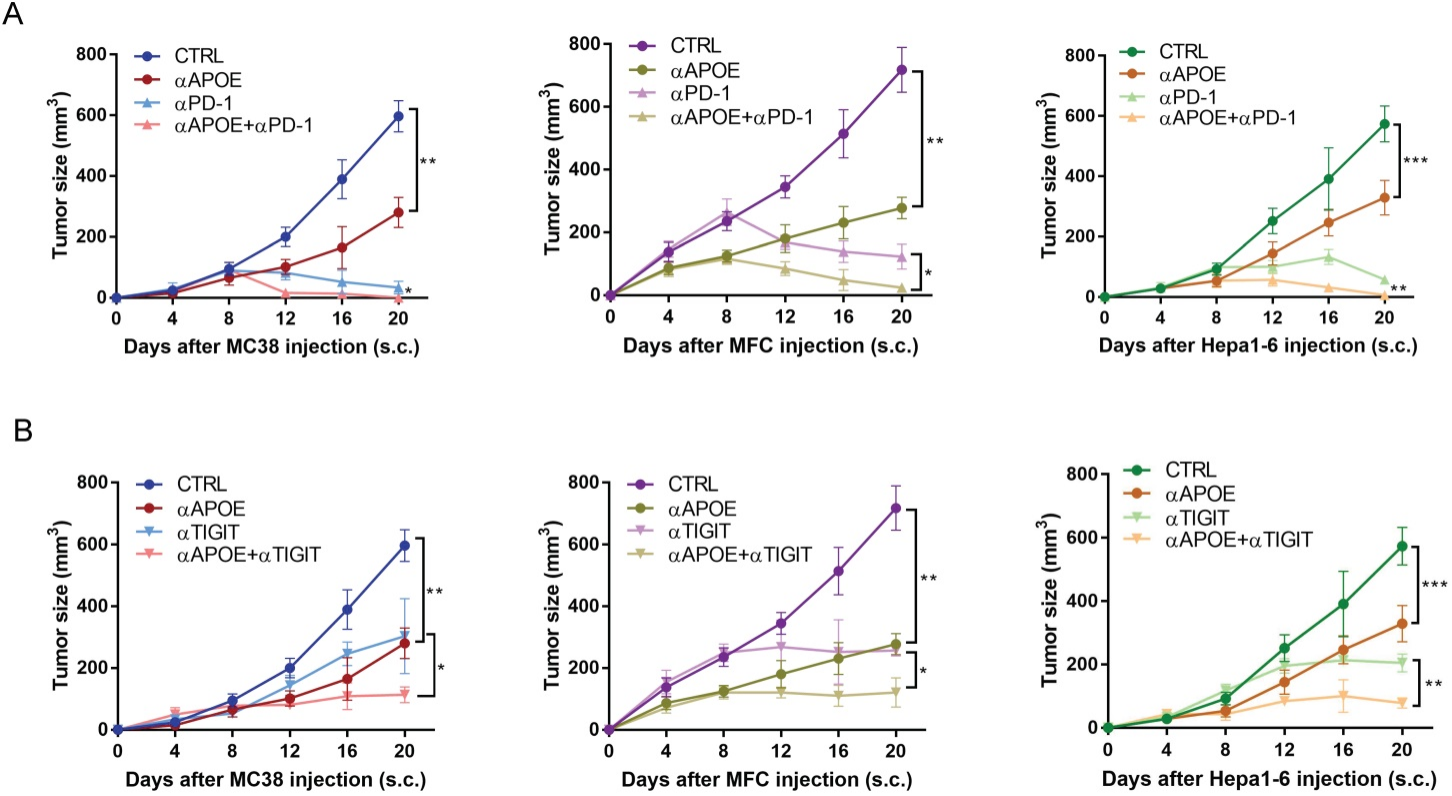
** The combination of APOE inhibitor and αPD-1/αTIGIT was an effective treatment for tumor. (A-B)**Tumor growth in mice injected subcutaneously with MC38, MFC, Hepa1-6, treated with αAPOE and αPD-1/αTIGIT. *p < 0.05, **p < 0.01, ***p < 0.001.

We examined αAPOE *in vivo* with or without αPD-1/αTIGIT for cancer therapy. αAPOE treatment was initiated at day two after the tumor injection and was repeated per five days until the end of the experiment. Treatment by using αAPOE alone achieved a significant but incomplete control of tumor growth. However, the combination of αAPOE and αPD-1/αTIGIT led to a complete tumor control in all mice tested (Figure 7A-7B). In brief, αAPOE had anti-tumor activity and augmented αPD-1/αTIGIT checkpoint blockade.

### APOE was negatively correlated with the expression of PD-1/TIGIT, and indicated the sensitivity of immunotherapy in cancers

**Figure 8:**
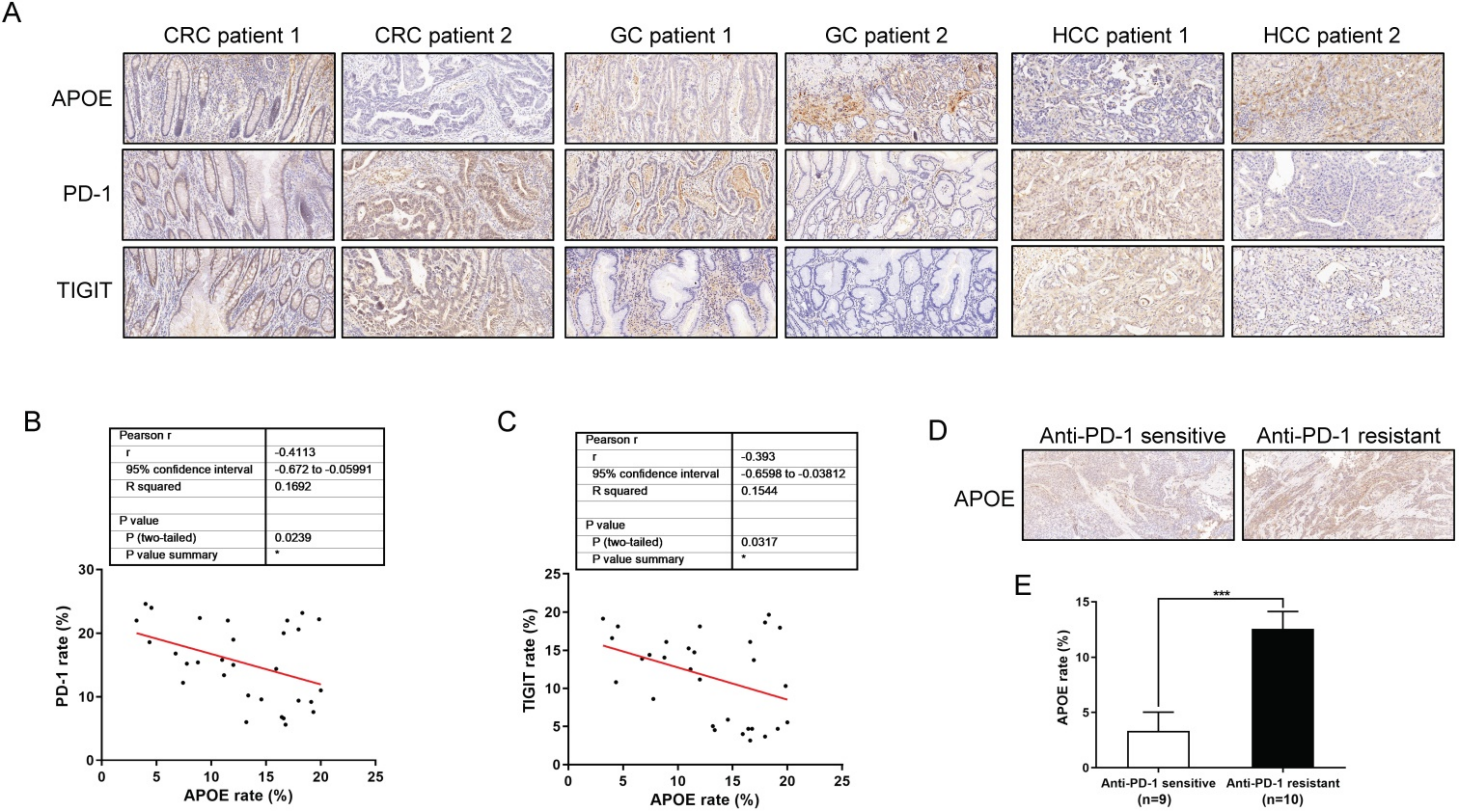
** APOE was negatively correlated with the expression of PD-1/TIGIT, and indicated the sensitivity of immunotherapy in cancers. (A-C)** Analysis of the correlation between APOE and the expression of PD-1 and TIGIT in 30 cancer patients. The figure (A) showed typical cases of three types of cancer. Figure B and C showed the correlation of PD-1 and TIGIT with APOE. **(D-E)** Analysis of APOE expression in PD-1 sensitive and resistant cancer patients.Patients were divided into two groups according to the RECIST 1.1 criteria. *p < 0.05, ***p < 0.001.

To further analyze the significance of the findings here in human tumors, we investigated APOE, PD-1 and TIGIT protein expressions from 30 human tumor specimens by using immunohistochemistry. Specific patient clinical information was shown in Table S2. As indicated from the correlation analysis, the expression of APOE showed a negative correlation with PD-1 and TIGIT in carcinomas (Figure 8A-8C). In addition, we examined the pre-treatment puncture samples from 19 carcinoma patients clinically treated with αPD-1, of whom 9 patients were αPD-1 sensitive and 10 patients were αPD-1 resistant (Table S3, S4). As revealed from the results, the expression of APOE in the αPD-1-resistant group significantly exceeded that in the αPD-1 sensitive group (Figure 8D-8E). Altogether, APOE might be a marker to predict the sensitivity of αPD-1, and its inhibitor could be used as a therapeutic means to enhance the sensitivity of αPD-1/αTIGIT checkpoint blockade.

## Discussion

This study demonstrated that constitutive lack of APOE or APOE inhibitor treatment curbed tumor growth.This is not the first time it has been reported in cancer. According to Kemp Samantha B et al. 10, orthotopic implantation of mouse pancreatic ductal adenocarcinoma (PDA) cells into syngeneic wild type or in Apoe^-/-^ mice showed reduced tumor growth in Apoe^-/-^ mice. Lee Yong Sun et al. 11 found that urethane-induced lung tumor incidence and B16F10 lung metastasis in Apoe^-/-^ mice were significantly reduced in comparison to that in WT mice. Buss Linda A et al. 12 reported that Apoe^-/-^ mice also had a reduced rate of metastasis compared to WT mice implanted with EO771 murine breast cancer cells. These results confirm that APOE knockout can indeed induce tumor reduction. However, one of best highlight of this research is that we used APOE inhibitor-COG 133TFA for cell and mice verification in cancer for the first time. COG 133 TFA competes with the APOE holoprotein for binding the LDL receptor, with potent anti-inflammatory and neuroprotective effects 13, 14. Previous study indicated that APOE induced the expressions of CXCL1 and CXCL5 by pancreatic tumor cells via LDL receptor and NF-κB signaling 10. Therefore, in this study, we innovatively tried to use COG 133TFA to explore its role in TAM cells. To our excitement, COG 133TFA in TAM reduced the proliferation, invasion and migration of cancer cells. In addition, treatment by using COG 133TFA *in vivo* alone achieved a significant control of tumor growth.

Mass spectrometry enables accurate analysis of different cell populations and comprehensive and accurate study of cytokines and signaling pathways simultaneously. In this study, we found that monocytes, NK cells, CD4^+^ T cells and CD8^+^ T cells as well as PD-1^+^ and TIGIT ^+^ cells were up-regulated whereas M2 macrophages were decreased in Apoe^-/-^ mice via mass spectrometry. The reason might be that the loss of APOE led to the reduction of M2 macrophages and thus reshaped the tumor immune microenvironment, which is more conducive to killing cancer cells. αPD-1/αTIGIT enhanced the antitumor effect in Apoe^-/-^ mice *in vivo*. Although APOE knockout caused the increase of exhaustion factors such as PD-1, TIGIT, the increase of CD8^ +^ T cell exhaustion was not enough to resist the killing effect of the remodeled new anti-cancer immune microenvironment, and αPD-1/αTIGIT combined with inhibition of APOE could completely eliminate cancer. Kemp Samantha B's study had also used mass spectrometry to confirm an increase in CD8^+^ T cells in tumors in Apoe^-/-^ mice 10. However, Tavazoie Masoud F's study showed that liver-X nuclear receptor (LXR) /ApoE activation therapy elicited robust anti-tumor responses and enhanced T cell activation during various immune-based therapies 15. The reasons for the different research results are as follows: 1) The objects of the study are different. Tavazoie Masoud F's study focused on myeloid-derived suppressor cells (MDSC), while ours focused on TAM. APOE may have different effects in different cells and different tumors. 2) Tavazoie Masoud F's study emphasizes the anticancer effect of LXR agonist combined with αPD-1 and APOE only explains part of the mechanism, while our study emphasizes the anticancer effect of APOE inhibitor COG 133 TFA combined with αPD-1. Ostendorf Benjamin N et al. 16 demonstrated that APOE4 mice showed enhanced antitumor immune activation relative to APOE2 mice, and T-cell depletion assays suggested that the effect of APOE genotype on melanoma progression was mediated by altered antitumor immunity. Melanoma patients with the APOE4 variant had improved survival compared with APOE2 carriers. APOE4 mice also showed improved outcomes in response to PD1 immune checkpoint blockade relative to APOE2 mice, and patients carrying APOE4 experienced improved anti-PD1 immunotherapy survival after progression of the first-line regimen. Our study did not classify APOE germline variants in detail, but supported this conclusion with general Apoe^-/-^ mice. The regulation mechanism of APOE *in vivo* might be very complex, and we believe that Kemp Samantha B's study research fits in well with our research for APOE on tumor immunity.

From the perspective of macrophages, we clarified that APOE knockout led to the decrease in M2 macrophages, especially C1QC ^+^ and CCR2 ^+^ macrophage cell population based on scRNA-seq, which presented novel insights into immunocombination therapy. The results were further confirmed by immunohistochemistry in three types of cancer. In addition, we stimulated cancer cells with TAM supernatant and found that macrophages in the αAPOE group significantly inhibited the proliferation, invasion and migration of cancer cells compared with the control group by CCK8, transwell and scratch assay. However, ALI EL ROZ et al. reported that inferior medium from APOE medium (“THP-1+siRNA”) was incubated with MCF-7 cells and compared with APOE-rich medium (“THP-1+LXR agonist”). MTT analysis showed that the viability of MCF-7 cells was reduced by 30% after 24 h incubation with “THP-1+LXR agonist” medium compared with control cells, whereas the viability of “THP-1+siRNA” medium was increased by 40% after 24 h %, with little effect after 48 h incubation. Interestingly, MCF-7 cell viability also decreased by 18% and 40% after 24 and 48 h incubation with exogenous APOE protein 17. Our results are completely contrary to the reports, which may be due to the different mechanism of action of APOE in different cell lines, or it may be that we are using APOE inhibitors rather than siRNA. The expression of APOE was also examined in the pre-treatment puncture samples of 19 carcinoma patients having received clinical αPD-1 treatment, and as revealed from the results, the expression of APOE in the αPD-1 resistant group significantly exceeded that in the αPD-1 sensitive group. This result presented sufficient evidence to elucidate the efficacy of APOE in humans.

Our research also has some shortcomings. First, we did not use myeloid specific APOE knockout mice to further verify the mechanism of APOE action in mouse tumor macrophages, because the culture time of knockout mice is too long. Second, our research is limited to tumors of digestive tract system, not cancer of other systems, and we look forward to subsequent research. Third, there are too few human samples to study, so it is expected to be confirmed by more human samples.

## Conclusion

**Figure 9:**
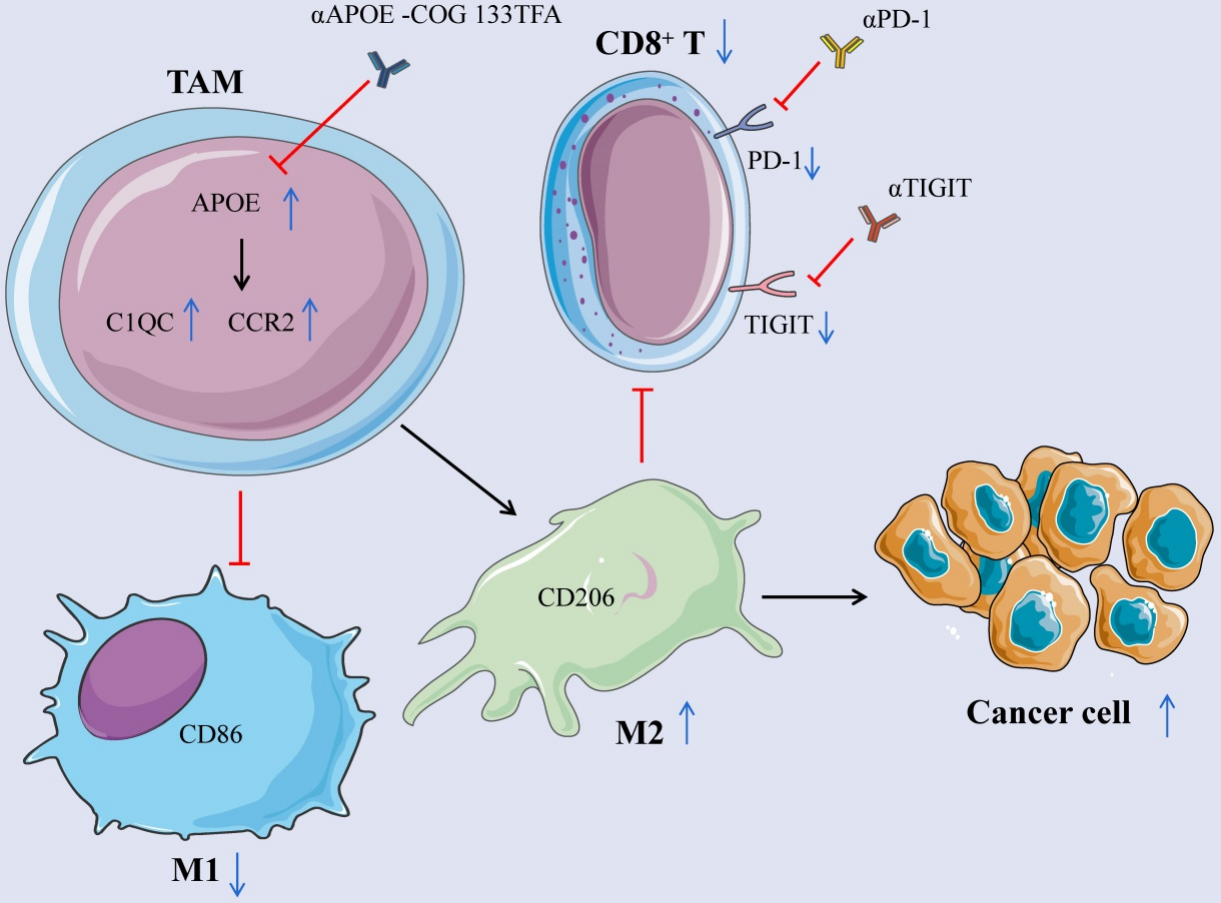
** Mechanism schema diagram.** The elevation of APOE in TAM induces the differentiation from M1 to M2 macrophages by promoting the expression of C1QC and CCR2, inhibits the expression of CD8^+^ T cells, reduces the expression of PD-1 and TIGIT on the surface of CD8 ^+^ T cells, and promotes the growth of cancer cells. Inhibition of APOE can induce the differentiation of M2 into M1 macrophages and increase the expression of CD8^+^ T cells. Meanwhile, αPD-1/αTIGIT combined with αAPOE -COG 133TFA can enhance the anti-tumor sensitivity.

APOE is an oncomarker of tumor-infiltrating macrophages in mouse models and human tumors. Its inhibitor COG 133TFA can slow down tumor growth, improve the therapeutic effect of checkpoint blocking, and reshape tumor immune microenvironment in cancers (Figure 9).

## Supplementary Material

Supplementary figures and tables.
