# Supplementary material for: Inhibition of APOE potentiates immune checkpoint therapy for cancer
Source: Int J Biol Sci. 2022 Aug 15;18(14):5230–40. doi: 10.7150/ijbs.70117 (PMC9461658; doi:10.7150/ijbs.70117)

Table S1. A summary of Software used for data analysis

Software	Source
R	https://www.r-project.org
Bioconductor	https://www.bioconductor.org
Cell Ranger	https://support.10xgenomics.com/single-cell-gene-expression/software
Seurat	https://satijalab.org/seurat
COATES	https://www.gsea-msigdb.org/gsea/msigdb/cards/COATES_MACROPHAGE_M1_VS_M2_DN

Table S2. Clinical information of 30 patients related to Figure 8A

**Patients**	**Gender**	**Age(years old)**	**Tumor types**	**Maximum tumor size(cm)**	**AJCC stage**
C1	Female	73	CRC	3.5	II
C2	Male	40	CRC	3.5	III
C3	Male	57	CRC	3	III
C4	Male	55	CRC	3	II
C5	Male	63	CRC	4.5	III
C6	Male	66	CRC	2.5	III
C7	Female	37	CRC	4	III
C8	Male	34	CRC	3.5	III
C9	Male	60	CRC	5	III
C10	Male	52	CRC	4.5	II
G1	Female	55	GC	2.5	II
G2	Male	70	GC	6.5	III
G3	Male	58	GC	2.7	III
G4	Male	66	GC	4	III
G5	Male	33	GC	8	III
G6	Male	64	GC	2	I
G7	Male	40	GC	1	III
G8	Male	66	GC	4	III
G9	Female	67	GC	5	III
G10	Male	52	GC	8.5	III
H1	Male	75	HCC	6.5	II
H2	Female	77	HCC	2.3	I
H3	Male	54	HCC	5.5	III
H4	Female	69	HCC	3.5	II
H5	Male	60	HCC	17	III
H6	Male	75	HCC	6.5	II
H7	Female	60	HCC	7	II
H8	Male	57	HCC	2	I
H9	Male	62	HCC	6.5	II
H10	Male	47	HCC	4.6	I

Table S3. Clinical information of 19 patients related to Figure 8D

Patients	Gender	Age(years old)	Tumor types	Maximum tumor size(cm)	AJCC stage	Sensitive (Yes or No)
PC1	Male	72	CRC	4	IV	Yes
PC2	Male	61	CRC	5	IV	Yes
PC3	Male	58	CRC	5.2	IV	Yes
PC4	Female	52	CRC	2.8	IV	Yes
PC5	Male	47	CRC	7	IV	Yes
PC6	Male	65	CRC	5	IV	Yes
PC7	Male	51	CRC	7	IV	No
PC8	Male	66	CRC	2.6	IV	No
PC9	Male	58	CRC	4	IV	No
PC10	Female	39	CRC	5.5	IV	No
PC11	Male	42	CRC	3	IV	No
PC12	Female	61	CRC	4.5	IV	No
PC13	Male	70	CRC	5	IV	No
PC14	Female	67	CRC	5.2	IV	No
PG1	Female	65	GC	3	IV	Yes
PG2	Female	72	GC	2	IV	No
PH1	Male	70	HCC	3.3	II	Yes
PH2	Male	55	HCC	8	III	Yes
PH3	Male	45	HCC	10.1	III	No

Table S4. Correlations of Anti-PD-1 response and clinical characteristics of 19 cancer patients

Variables	Anti-PD-1 sensitive (n = 9)	Anti-PD-1 resistant (n = 10)	p value
**Gender**			0.40515
Male	7	6	
Female	2	4	
**Age**			0.80808
≤60	4	5	
＞60	5	5	
**Tumor size**			0.76418
≤4cm	3	4	
＞4cm	6	6	
**TNM stage**			0.27879
I/II	1	0	
III/VI	8	10	
**Distant metastasis**			0.46577
No	2	1	
Yes	7	9	

Figure S1: Mass spectrometry flow processing steps.

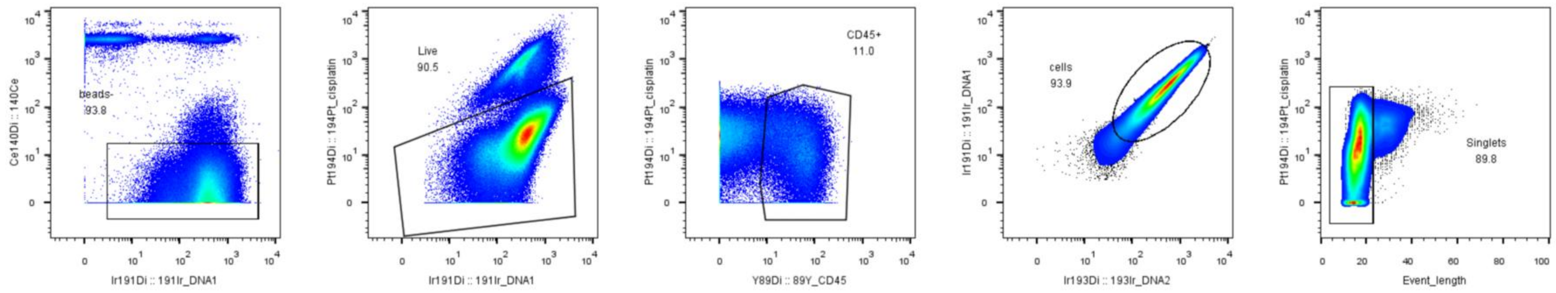

Figure S2: TSNE diagram showing the marker gene expression in mass spectrometry process.

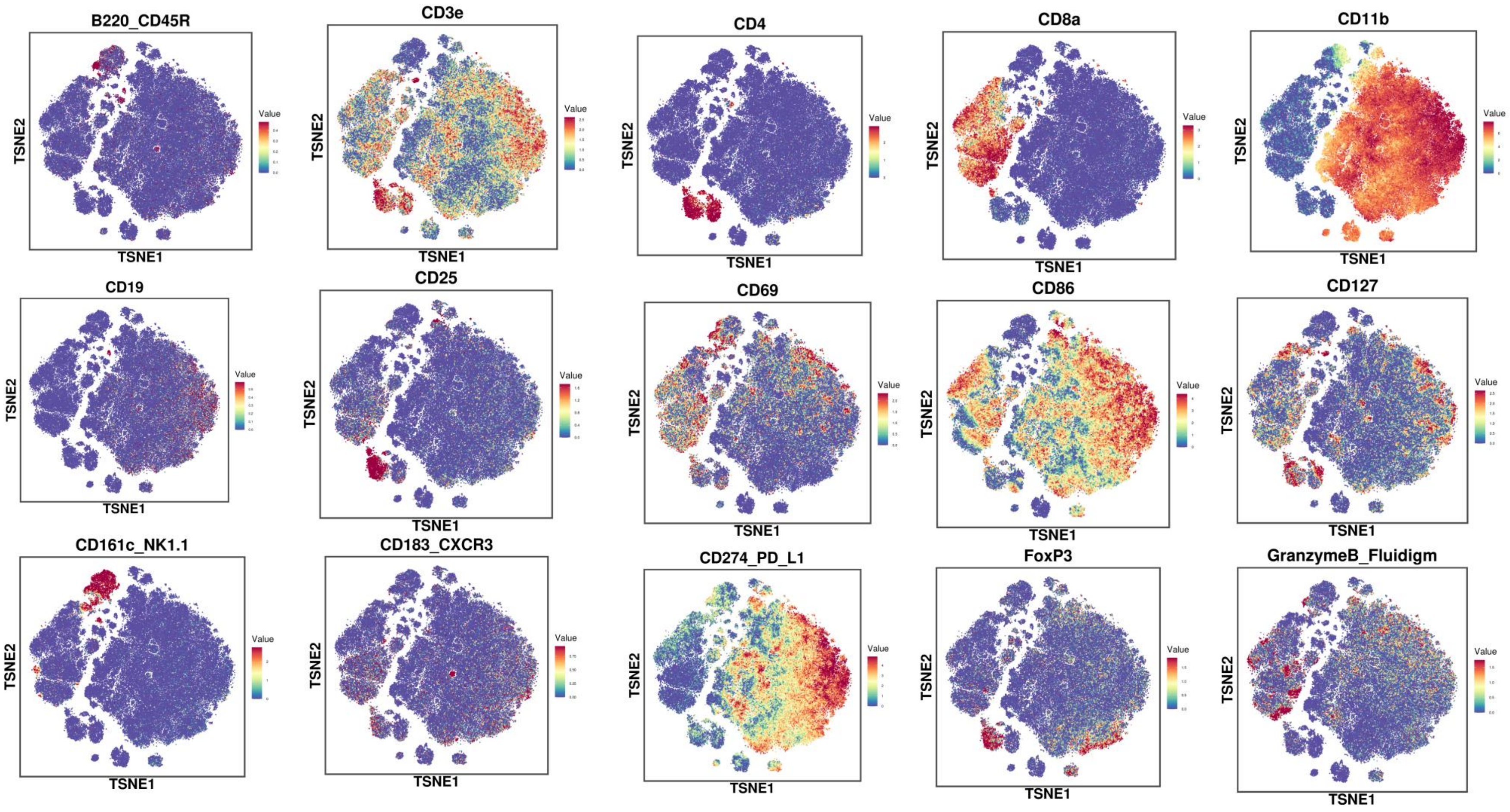

Figure S3: (A) Immunohistochemistry was used to confirm the expression of C1QC and CCR2 in tissues of the three cancer models with Apoe$^{+/+}$ and Apoe$^{-/-}$ mice. (B) Immunohistochemistry was used to confirm the expression of CD86, CD206, C1QC and CCR2 in spleens of Apoe$^{+/+}$ and Apoe$^{-/-}$ mice without injection of cancer cells.

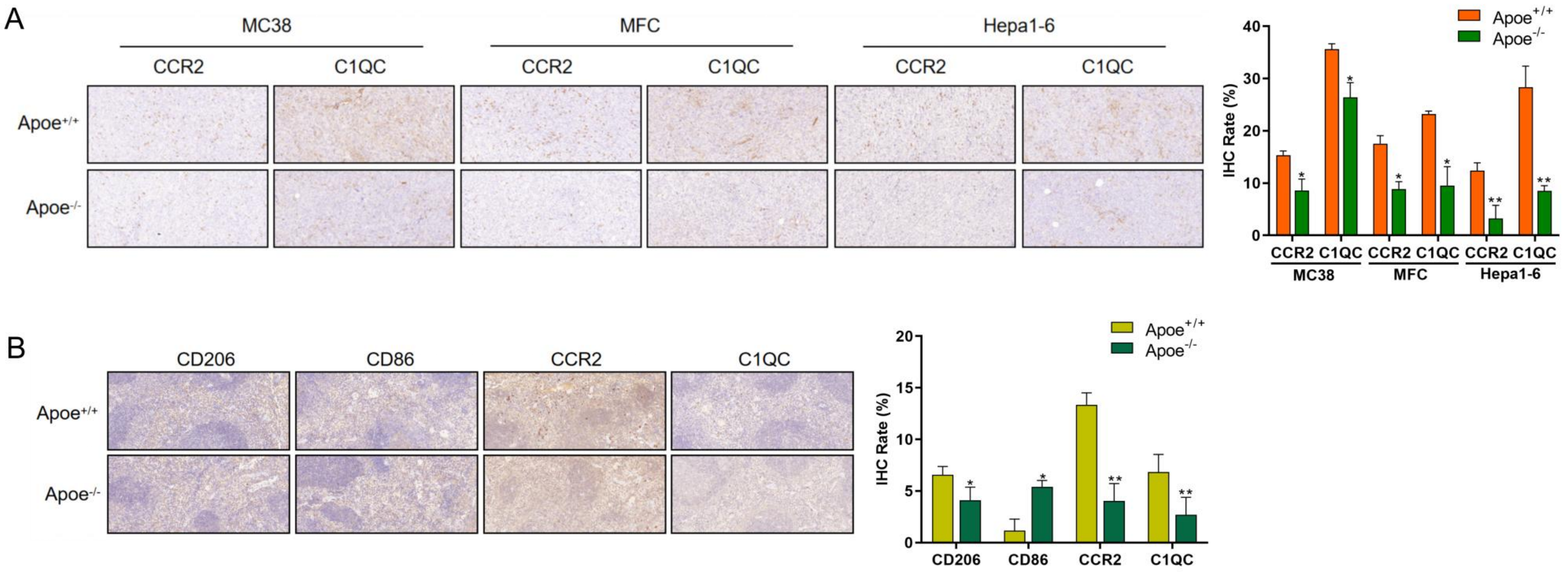

Supplement: Supplementary file 1 — Supplementary figures and tables. [file ijbsv18p5230s1.pdf]
